# Magnetic resonance imaging of a randomized controlled trial investigating predictors of recovery following psychological treatment in adolescents with moderate to severe unipolar depression: study protocol for Magnetic Resonance-Improving Mood with Psychoanalytic and Cognitive Therapies (MR-IMPACT)

**DOI:** 10.1186/1471-244X-13-247

**Published:** 2013-10-05

**Authors:** Cindy C Hagan, Julia ME Graham, Barry Widmer, Rosemary J Holt, Cinly Ooi, Adrienne O van Nieuwenhuizen, Peter Fonagy, Shirley Reynolds, Mary Target, Raphael Kelvin, Paul O Wilkinson, Edward T Bullmore, Belinda R Lennox, Barbara J Sahakian, Ian Goodyer, John Suckling

**Affiliations:** 1 Department of Psychiatry, University of Cambridge, Herchel Smith Building for Brain and Mind Sciences, Robinson Way, Cambridge CB2 0SZ, UK; 2 Department of Psychiatry, University of Cambridge, Douglas House, 18b Trumpington Road, Cambridge CB2 8AH, UK; 3 Psychoanalysis Unit, Research Department of Clinical, Educational and Health Psychology, University College London, Gower Street, London WC1E 6BT, UK; 4 Department of Psychological Sciences, Norwich Medical School, University of East Anglia, Norwich NR4 7QH, UK; 5 Brookside Family Consultation Clinic, 18d Trumpington Road, Cambridge CB2 8AH, UK; 6 MRC/Wellcome Trust Behavioural and Clinical Neurosciences Institute, University of Cambridge, Herchel Smith Building for Brain and Mind Sciences, Robinson Way, Cambridge CB2 0SZ, UK; 7 Department of Psychiatry, University of Oxford, Warneford Hospital, Oxford OX3 7JX, UK; 8 Cambridgeshire and Peterborough NHS Foundation Trust, Cambridge, UK; 9 GlaxoSmithKline, Clinical Unit Cambridge, Cambridge, UK

## Abstract

**Background:**

Major depressive disorders (MDD) are a debilitating and pervasive group of mental illnesses afflicting many millions of people resulting in the loss of 110 million working days and more than 2,500 suicides per annum. Adolescent MDD patients attending NHS clinics show high rates of recurrence into adult life. A meta-analysis of recent research shows that psychological treatments are not as efficacious as previously thought. Modest treatment outcomes of approximately 65% of cases responding suggest that aetiological and clinical heterogeneity may hamper the better use of existing therapies and discovery of more effective treatments. Information with respect to optimal treatment choice for individuals is lacking, with no validated biomarkers to aid therapeutic decision-making.

**Methods/Design:**

Magnetic resonance-Improving Mood with Psychoanalytic and Cognitive Therapies, the MR-IMPACT study, plans to identify brain regions implicated in the pathophysiology of depressions and examine whether there are specific behavioural or neural markers predicting remission and/or subsequent relapse in a subsample of depressed adolescents recruited to the IMPACT randomised controlled trial (Registration # ISRCTN83033550).

**Discussion:**

MR-IMPACT is an investigative biomarker component of the IMPACT pragmatic effectiveness trial. The aim of this investigation is to identify neural markers and regional indicators of the pathophysiology of and treatment response for MDD in adolescents. We anticipate that these data may enable more targeted treatment delivery by identifying those patients who may be optimal candidates for therapeutic response.

**Trial registration:**

Adjunctive study to IMPACT trial (Current Controlled Trials: ISRCTN83033550).

## Background

Major depressive disorders (MDD) are serious public and individual mental health illnesses predicted to be the leading health burden worldwide by 2030 [1]. First episodes of MDD often occur during childhood or adolescence, with additional episodes likely to recur in some 65% of cases [2,3]. Further to this, Harrington and Clark [4] report that childhood or adolescence onsets of affective disorder are evident in approximately 30% of adult cases. Adolescence can therefore be considered a critical period where the initial stages and trajectories of depression emerge [5]. Prior history of depression is associated with an increased risk of recurrence of MDD irrespective of concomitant level of prior or subsequent environmental adversity [6,7] indicating a key role for the illness per se in influencing natural history within individuals and making the delivery of effective treatment at the earliest stages of the illness highly important in relapse prevention.

Tackling this debilitating illness most effectively at the first emergence of an episode in adolescence could be seen as a crucial prevention strategy that may reduce the risk of recurrence of future episodes and prove integral for the long-term improvement of individual health outcomes. In this respect, structural and functional neuroimaging measures, either alone or in combination, have been used to identify brain areas indicative of efficacious treatment given in adulthood [8-10]; reviewed in [11,12]. Furthermore, structural and functional neuroimaging measures could have important prognostic clinical potential by identifying those individuals who are most likely to benefit from one or more forms of treatment [8,11].

### Treatments for MDD in adolescence

The United Kingdom (UK) National Institute for Health and Care Excellence (NICE) guidelines recommend a stepped-care approach for patients with MDD, where treatment options advance alongside escalating need for care. Although not always successful in leading to remission, provision of treatment, either in the form of psychotherapy, pharmacotherapy, or both, often is essential in order to interrupt the cycle of episodic relapse and recurrence in MDD. In adolescents with MDD, a form of psychotherapy such as cognitive behavioral therapy (CBT) is first provided which may or may not be accompanied by pharmacotherapy treatment with selective serotonergic reuptake inhibitors (SSRIs) such as fluoxetine (20 mg-60 mg).

A pragmatic randomised controlled superiority trial by our group compared SSRI and specialist clinical care (SCC) with a combined treatment regime of SCC + SSRI + CBT and showed that the addition of CBT was no more effective than SCC + SSRI at effecting remission by 28 weeks of treatment [13]. Although that study did not have a CBT-only or non-treatment control group, March and colleagues [14] noted that CBT alone was not superior to placebo in effecting remission by 12 weeks and both were less effective than fluoxetine alone or combination therapy.

### Brain imaging studies of MDD in adults

The IMPACT and MR-IMPACT research programmes aim to establish the extent to which psychotherapies with or without fluoxetine diminish the risk for relapse at 86 weeks after treatment and reveal neural regions or patterns of activity associated with treatment remission, recovery or relapse. A meta-analysis conducted by Fu and colleagues [11] compared neuroimaging evidence for volumetric and functional changes associated with symptom improvement following treatment (pharmacological, psychological, and combined approaches) for depression. Functional activity in bilateral regions of pregenual anterior cingulate cortex (pgACC) and right subgenual anterior cingulate cortex (sgACC)/medial orbitofrontal cortex (OFC) positively correlated with treatment response, with greater activity in those regions associated with an increased likelihood of responding to treatment for depression [11], mirroring findings from an earlier study [8]. A negative correlation between activation in right anterior insula and right putamen and treatment response also was observed [11]. Notably, a recent report by McGrath and colleagues [15] suggested that metabolic activity in right anterior insula could differentiate pharmacological versus psychological treatment response in patients with moderate to severe depression with the potential to optimize initial treatment selection in MDD.

In terms of brain structural predictors of treatment response, regional volume reductions in the right hippocampus, as determined through manual segmentation techniques, were associated with poorer response to treatment [11]. Although lacking sufficient power, reduced volume in bilateral dorsolateral prefrontal cortex (dlPFC), a region involved in the cognitive regulation of emotion, was associated with poorer response to treatment for depression. It is noteworthy that a recent meta-analysis of treatment effects in MDD observed across fMRI studies also identified bilateral dlPFC as a treatment-sensitive region [16]. Considered together, treatment response in MDD appears to be predicted by a number of key areas within the fronto-limbic network [17-19] including pgACC, sgACC, hippocampus, and prefrontal cortices. Increased activity in perigenual ACC (pgACC and sgACC regions) has been linked to successful treatment by pharmacological agents whereas decreased activity in this region has been linked to successful treatment by psychological therapy [8,9,20]; summarized in [12].

While clear support can be garnered for a fronto-limbic pathophysiology of MDD, disruption to other neural and neuroendocrine circuits such as the hypothalamic-pituitary-adrenal (HPA-axis), cortico-striato-pallidal-thalamo-cortical (CSPTC) loop, default mode network, and reward network (further discussed in [12,16,21,22]) have been discussed in relation to MDD. Following CBT treatment significant interaction effects have been observed in the amygdala and dorsal ACC for patients with MDD compared to healthy control participants [20] with treatment normalizing the previously elevated and reduced responding in these respective regions. Interestingly, the meta-analysis by our group [16] showed *decreased* functional activation in the amygdala in adults with MDD relative to healthy controls, perhaps indicating that the common notion of an over-active limbic system in MDD is incorrect or that the treatment strategies formerly undertaken by adults with MDD elicit considerably sustained effects in the amygdala. Dorsal ACC is not part of the fronto-limbic network [19] but may modulate both reward and HPA axis networks [22,23], suggesting the interaction of multiple neural systems in MDD [24]. In line with a multiple systems approach, the amygdala is part of the fronto-limbic circuit and CSPTC loop [16,19], and interacts with the HPA-axis [21], thus disruption to this region alone could lead to perturbations downstream affecting many neural systems. Different subtypes of depression (some of which remain to be identified) could also be associated with impairment to different neural systems, or with disruption to multiple neural systems at varying weights.

### Brain imaging studies of MDD in adolescence

Given the aberrant patterns of brain structure and function observed in adults with MDD, we can ask whether depression in adolescence is similar to depression in adulthood and whether common neural mechanisms underlie pharmacological and/or psychological effectiveness in spite of developmental processes occurring in the adolescent brain. Far fewer neuroimaging investigations of depression have been conducted in adolescents, with no single meta-analysis performed to our knowledge. Recent studies, however, suggest similar perturbations in brain structure and function in regions including, but not limited to, dlPFC, OFC, sgACC, and amygdala, irrespective of patient age [25-27]. Furthermore, fluoxetine may exert effects via common neural mechanisms in both adolescents and adults with MDD [26], and CBT treatment response was unaffected by age in a small group of adolescents with MDD [28]. These neuroimaging studies together suggest that MDD and effective treatment thereof may be instantiated by comparable mechanisms in adolescents and adults.

### Limitations of neuroimaging studies of MDD

There are, however, several limitations of previous neuroimaging research studies of MDD. Often mixed are cases that may represent distinct subtypes of MDD hence limiting the generalizability of findings. Furthermore, many treatment investigations of MDD have not examined treatment effects past 16 weeks, which is a relatively short time window considering that recovery may take up to 2 years in some cases [29], and that remission rates are 50% in first episode cases of clinic groups by 2 years [29]. Longitudinal investigations across the entire period of recovery are needed to understand why some individuals recover from MDD whereas others do not. The sample sizes of many neuroimaging studies are relatively small considering the substantial clinical heterogeneity across MDD cases. A failure to find statistically significant differences across the whole-brain may have prompted some studies to investigate case–control differences within hypothesis-driven regions of interest (ROI), which apply more lenient criteria for evaluating the statistical significance of results. When coupled with a relatively underpowered sample, inadvertent consequences of adopting ROI approaches are the potential to inflate Type I and Type II error rates due to the possibility of scanning an unrepresentative sample of MDD cases and a failure to include all regions where significant differences may occur across groups or conditions. Larger sample sizes permitting whole-brain examinations of volumetric and functional activation differences are therefore needed in order to better understand the pathophysiology of depression in adolescence.

### Aims

Neuroimaging examinations of adolescents with MDD provide an opportunity to reveal and differentiate pathophysiological and therapeutic neural structure and function between first episode sporadic as well as at risk recurrent forms of this disorder. Using a repeated-measures design in selected cases that have completed psychological treatment provides a longitudinal neuroimaging investigation of therapeutic effects that have the added potential of revealing neural sites that constitute state or trait markers of illness and treatment insensitivity.

MR-IMPACT will therefore examine neural markers of moderate to severe unipolar depression in adolescence and neurobiological predictors of recovery from depression. Specific objectives of MR-IMPACT are as follows:

• To define the profile of brain structural and functional differences in adolescent patients with depression at baseline compared to a group of healthy adolescent participants.

• To assess baseline measurements of brain structure, functional activation and connectivity, focusing on neural systems implicated in adult MDD (ACC and other fronto-limbic components) as predictors of symptomatic response to each of the three types of psychological treatments in the short-to-medium term (up to 86 weeks after the start of treatment).

• To measure changes in brain functional activation and connectivity before and after a course of CBT and development-related changes in brain function measured in a comparison group of healthy participants to permit identification of treatment-related changes in brain function.

## Methods/Design

### Study overview

**Figure 1:**
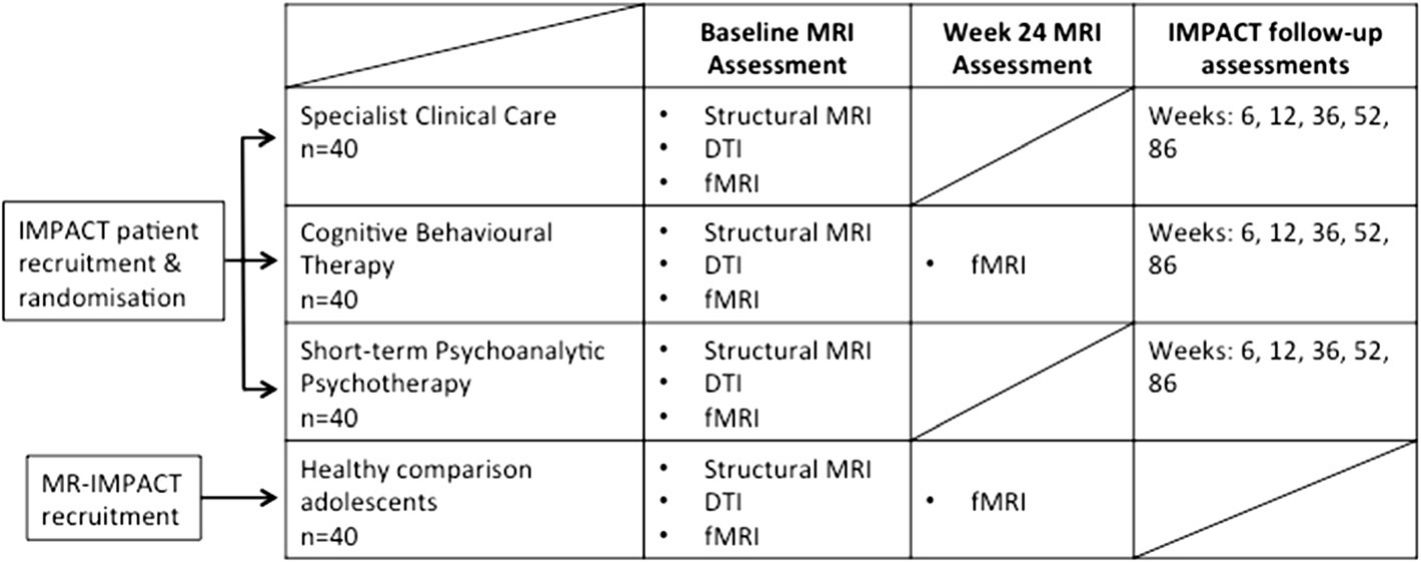
The relation of the IMPACT trial to the MR-IMPACT study.

MR-IMPACT is a pragmatic effectiveness trial of depression in adolescence. All adolescents enrolled in MR-IMPACT are simultaneously taking part in the Improving Mood with Psychoanalytic and Cognitive Therapies (IMPACT) trial [30], and have been randomly allocated to one of three forms of talking therapy (short-term psychoanalytic psychotherapy [STPP], SCC, and CBT; Figure 1). The IMPACT recruitment, baseline assessment and randomization procedures can be found together with the treatment protocol for each therapeutic arm in Goodyer et al. [30]. Another adjunct study of IMPACT, IMPACT-genes and hormones, will collect salivary cortisol pre- and post-therapy to assess HPA-axis functioning [30]. In adherence to UK NICE guidelines, patients allocated to any treatment arm may also be prescribed up to 50 mg of Fluoxetine for symptom reduction in the presenting episode.

The study will be conducted in accordance with the Declaration of Helsinki [31].

### Study participants

The patient population will be recruited from a larger cohort of young people with depression who have agreed to take part in the IMPACT trial. All eligible and interested patients residing in London or Cambridgeshire will be invited to take part in MR-IMPACT, with enrolment targeted to reach 120 adolescents (male and female) aged 11 through 17 years who meet Diagnostic and Statistical Manual of Mental Disorders (DSM-IV) criteria for moderate to severe unipolar depression. Patients with chronic or recurrent MDD are also included, as are patients already taking antidepressant medication. A detailed list of the study exclusion criteria is provided below.

All patients will be provided an MR-IMPACT study information leaflet and will provide verbal consent to IMPACT research assistants to be contacted by telephone by an MR-IMPACT researcher a minimum of 48 hours later for potential participation in the MR-IMPACT study. Patients will be contacted to assess interest in and eligibility for the magnetic resonance imaging (MRI) scan. Patients with MRI contraindication will be ineligible and therefore excluded from the study at this point. For eligible patients, if the patient so chooses, one adult (typically a parent) may accompany the adolescent patient into the scanner suite. For the patients requesting this option, the identified adult will be screened over the telephone for MRI contraindications. Eligible patients will be scheduled for an MRI appointment where they are remunerated £30 for their time and inconvenience. Any fees incurred for travel to and from the MRI appointment will be reimbursed to the adult accompanying the patient.

A group of healthy control young persons matched for age, sex, intelligence quotient (IQ) and handedness will be recruited into the study for comparison. A letter will first be posted to all families with healthy adolescents who had participated in a previous study (i.e. the ROOTS study) [32]. The letter will describe the MR-IMPACT study and the current recruitment of siblings of ROOTS participants aged between 11 and 17 years, and request that interested families contact a member of the MR-IMPACT team to discuss participation and arrange a home visit. If needed, additional healthy control adolescents will be recruited from local schools within Cambridgeshire.

Prior to visiting the home, potential healthy control families will be posted MR-IMPACT study information sheets at least 48 hours in advance to allow ample time to consider study participation. At the home visit the study details will be reviewed prior to obtaining informed assent and consent by potential participants and their parent/caregiver, respectively. Screening for personal and family history of psychiatric illness and MRI contraindication will then be conducted. In a private setting, healthy adolescents will also be screened for history of attempted suicide, suicidal thoughts or cutting behaviour. After the home visit eligible healthy adolescents and their families will be contacted to arrange an MRI appointment at their convenience where adolescents will be given a stipend of £30 for their time and inconvenience. Any expenses incurred for travel to and from the MRI appointment will be reimbursed to the adult accompanying the healthy adolescent.

### Screening and MRI assessment

At the MRI assessment adolescents and their parents will be asked to review the study information sheet one final time prior to signing the MR-IMPACT study assent and consent forms. Adolescents will be re-screened for presence of MRI contraindication, as will any adults requested to accompany adolescents in the scanner suite. Adolescents will then be asked to complete the Edinburgh Handedness Inventory [33] and the Spielberger Trait Anxiety Inventory [34]. In preparation for the tasks the adolescent will be asked to complete in the MRI scanner, three similar tasks brief in length will be practiced on a computer in advance of the actual scan. The practice tasks will be administered in the same order as the MRI tasks are presented to minimise confusion. Patients will then be asked to complete the Spielberger State Anxiety Inventory [34] before attending the 1.5 hour MRI session. Upon completion of the MRI session, patients will be asked to complete the Bond-Lader Visual Analogue Scales [35] and then the Mood and Feelings Questionnaire-Short version [36]. Lastly, patients will be asked to complete two computerised tasks in follow-up to one of the completed fMRI tasks.

### Eligibility criteria

All patient participants will be required to meet DSM-IV criteria for moderate to severe MDD, as determined by child and parent interviews using the Kiddie Schedule for Affective Disorders and Schizophrenia-Present and Lifetime version (K-SADS-PL) [37]. A second criterion for inclusion within the IMPACT trial is a score of 27 or more on the self-report Moods and Feelings Questionnaire (MFQ).

### Inclusion criteria

1. Aged 11 through 17 years.

2. Current DSM-IV unipolar MDD diagnoses with moderate to severe impairment determined by child and parent interviews using the K-SADS-PL.

3. Score of 27 or more on the MFQ.

### Exclusion criteria

1. Alcohol dependence.

2. Drug dependence.

3. Pervasive developmental disorder or generalized learning problem.

4. Pregnancy or breastfeeding.

5. Concurrent medication use that could adversely interact with SSRIs.

6. MRI contraindication.

7. Brain abnormality.

8. Intolerance to the MRI environment.

In order to ensure that the patient sample remains representative of the cases that undergo treatment through National Health Service Child and Adolescent Mental Health Services (NHS CAMHS), no further exclusions are made.

### Ethics

Ethical approval for the study was granted by the Cambridgeshire 2 Research Ethics Committee, Addenbrooke’s Hospital Cambridge, UK (REC reference: 09/H0308/168).

### Assessments and outcome measures

#### *Self-report battery*

**Edinburgh handedness inventory** The Edinburgh Handedness Inventory [33] assesses preferred hand use across different tasks and generates a single score ranging from -100 to +100 (i.e., the laterality quotient) indicative of overall handedness laterality. Across a series of tasks, participants are requested to indicate which hand typically performs said task. In the event that neither hand is the preferred hand, a score is evenly allocated to both left and right hands. The measure is valid for use across cultures and between sexes.

**Spielberger state-trait anxiety inventory** The Spielberger State-Trait Anxiety Inventory (STAI) [34] is comprised of two 20-item questionnaires that assess both temporary fluctuations in, and long-standing experience of anxiety symptoms. When completing the STAI-State (STAI-S), participants are asked to indicate whether each statement within a list of statements applies to them 'not at all’, 'somewhat so’, 'moderately so’, or 'very much so’ at that particular moment in time. Scores indicative of a high internal consistency are more valid than test-retest correlations when assessing the validity of transitory measures such as the STAI-S. The median alpha coefficient for the STAI-S was approximately 0.93, while the median test-retest correlation was 0.330 [34]. When completing the STAI-Trait (STAI-T), participants are asked to indicate how much each statement within a list of statements applies to them in general (almost never; sometimes; often; or almost always). The median alpha coefficient was 0.90 for the STAI-T scale indicating a high level of internal consistency, with test-retest correlations observed to be reasonably high across high school students, achieving a median-level of 0.695 [34].

The STAI-S anxiety questionnaire attempts to measure current (i.e., temporary) feelings of tension, apprehension, worry and nervousness whereas the STAI-T anxiety questionnaire attempts to measure long-standing individual differences in anxiety susceptibility. State anxiety scores can therefore be considered as reflecting the intensity of anxious feelings and trait anxiety scores can be considered as reflecting the frequency with which feelings of anxiety are experienced. Scores obtained from the STAI-S and STAI-T correlate positively with each other, particularly in the case of sequential administration.

**Bond-lader visual analogue scales** The Bond-Lader Visual Analogue Scales (VAS) is a highly sensitive questionnaire comprised of several rating scales that can detect subtle mood changes in a large sample of healthy individuals aged 16–64 years [35]; c.f. [38]. The VAS measures subjective feelings relative to 16 opposing emotive word-pairs. Emotive word-pairs are presented with one word appearing at each polar end of a single line measuring 100 mm. Participants are asked to indicate how they currently feel relative to the sixteen emotive dimensions by drawing a perpendicular line on each rating line. The length from the end of the line to the participant’s mark is then measured to provide an overall score for each of the 16 scales.

While items within the VAS were once thought to assess mental alertness, physical alertness, calmness, and other feelings [38], factor analysis of the measure in healthy individuals has since revealed only three underlying scales: alertness; contentedness; and calmness [35].

**Short mood and feelings questionnaire** The Short Mood and Feelings Questionnaire (SMFQ) [36] is a 13-item self-report questionnaire that measures the presence and severity of depressive symptoms occurring in an individual over a two-week period. The scale derives from the 33-item MFQ, with each item consisting of a single statement (e.g., 'I didn’t enjoy anything at all’) rated by the respondent as 'always true’ , 'mostly true’ , 'sometimes true’ , or 'never true’.

The SMFQ reviews the core set of depressive symptoms and has been shown to discriminate child psychiatric patients from pediatric controls, and depressed from non-depressed participants in a general population sample. The SMFQ correlates highly with more extensive evaluations, like the Children’s Depression Inventory (CDI) or the Diagnostic Interview Schedule for Children (DISC), and has a robust single factor structure from ages 6 to 16 years.

### MRI assessment battery

#### *Structural MRI datasets*

A three-dimensional structural MRI (sMRI) will first be obtained for all participants to exclude for brain abnormality. MR images will be acquired on a 3.0 Tesla Magnetom Trio Tim scanner (Siemens, Surrey, England) at the Wolfson Brain Imaging Centre, University of Cambridge, UK. High-resolution dual echo sequences will be acquired in the axial plane using proton density T2-weighted sequences (Axial PD-T2; 27 slices of 4.0 mm thickness, first echo time (TE) = 12.0 ms, second TE = 104.0 ms, repetition time (TR) = 4.60s, flip angle = 150˚, field of view = 224 × 134 mm^2^, voxel size = 0.7 × 0.7 × 4.0 mm^3^, series = interleaved). High-resolution T1-weighted sequences will be acquired in the sagittal plane using a three-dimensional magnetically prepared rapid acquisition gradient echo sequence (3D-MPRAGE; 176 slices of 1.0 mm thickness, TE = 2.98 ms, TR = 2.30s, inversion time = 900 ms, flip angle = 9˚, field of view = 240 × 256 mm^2^, voxel size = 1.0 × 1.0 × 1.0 mm^3^, series = interleaved). All images will be examined and approved for inclusion by a Consultant Radiologist specialising in neuroanatomy. Grey and white matter maps will be generated for each baseline scan.

#### *Diffusion tensor imaging (DTI)*

Whole brain data will be acquired and analysed for derivation of fractional anisotropy measures of white matter integrity and tractography of selected axonal tracts mediating connections between limbic and frontal regions of interest. Thirteen diffusion-weighted directed volumes and 5 volumes without diffusion-weighting (b = 0) will be acquired (63 slices of 2.0 mm thickness, TE = 98 ms, TR = 8.30s, field of view = 192 × 126 mm^2^, voxel size = 2.0 × 2.0 × 2.0 mm^3^, series = interleaved).

#### *Functional MRI (fMRI) datasets*

Echo-planar images (EPI) depicting blood oxygen level-dependent (BOLD) contrast at a sampling time of 2.0 seconds will be acquired during the performance of three tasks and during rest. MR images will be acquired on a 3.0 Tesla Magnetom Trio Tim scanner (Siemens, Surrey, England) at the Wolfson Brain Imaging Centre, University of Cambridge, UK. Radio frequency pulses will be transmitted and received via a quadrature birdcage headcoil. To maximise magnetic field homogeneity, an automatic shim will be applied prior to functional data acquisition. MR data covering the whole brain will be acquired using echo planar T2* weighted imaging. For each functional task, 32 slices of data 3.0 mm in thickness parallel to the anterior-posterior commissure will comprise each volume of data collected (TE = 30 ms, TR = 2 s, flip angle = 78˚, and interleaved series). The field of view and voxel size will be 192 × 120 mm^2^ and 3.0 × 3.0 × 3.0 mm^3^, respectively.

**Implicit facial expression recognition task** Facial expressions taken from the Facial Expressions of Emotion: Stimuli and Tests (FEEST) [39] representing medium and high intensity morphs of sadness and happiness are presented together with neutral faces and crosshair fixations which will serve as a low-level baseline to control for possible differences in activation of low-level visual areas. Facial expressions from four male and four female actors will be selected from the stimulus set based upon similarities in Facial Action Coding System unit score and high recognition ratings for happy, sad and neutral expressions. A total of 96 trials will be presented (16 from each category) with all stimuli presented in random order in an event-related design. A jittered stimulus presentation will be used with a mean inter-trial interval (ITI) of 3.0 seconds and a range of 1.0 to 6.0 seconds following a Poisson distribution. Each stimulus will appear on the screen for 2 seconds and for each facial trial adolescents will be asked to indicate the gender of the face by depressing one of two keys on a button box. All stimuli will be presented using Visual Basic software (Microsoft), with the order of stimulus presentation pseudo-randomized across participants to minimize habituation effects.

After each scan session participants will be asked to use a 9-point Likert scale, with 1 and 9 representing low and high intensities, respectively, to indicate how happy and how sad each of the emotional faces appear.

**fMRI data acquisition–implicit facial expression recognition** The scan length is approximately 8.5 minutes, during which 249 volumes of fMRI data will be collected.

**Emotional go/no-go task** Happy, sad and neutral words will be individually presented in a blocked paradigm design. The words presented are originally reported in Murphy et al. [40], with no word overlapping with the words used in the encoding and retrieval task described below. Within each block of presented words participants will be instructed to respond by button-press to words of a chosen target category (go), while withholding responses (no-go) to distracters selected from a different category. The task design will be similar to that reported in Elliott et al. [41], with each word block lasting 24 seconds in length and consisting of 10 targets and 10 distractor words. A total of 21 word blocks will be presented where six blocks of target sad words will be presented in the context of happy (3 blocks) and neutral (3 blocks) distractor words, six blocks of happy target words will be presented in the context of sad (3 blocks) and neutral (3 blocks) distractor words, and six blocks of neutral target words will be presented in the context of happy (3 blocks) and sad (3 blocks) distractor words. Three baseline word blocks consisting of italicised words (orthographic targets) presented in the context of plain text distracter words will also be presented. A 12 second rest block during which a crosshair fixation will appear for the first 8 seconds and simple task instructions (e.g., “Press for Sad words. Ignore Happy words.”) will appear for the remaining 4 seconds prior to each word block. Within every block, each word will be presented for 450 ms and will be followed by a gray screen ITI of 750 ms.

**fMRI data acquisition–emotional go/no-go task** The scan length is approximately 13.5 minutes, during which 399 volumes of data will be collected.

**Encoding and retrieval task** Anderson’s list of 555 personality trait words rated for likeableness [42] will be used to construct a list of 90 unambiguously positive and 90 unambiguously negative words. Participants will first be asked to perform an explicit categorisation (memory encoding) task and then an unanticipated incidental memory (retrieval) task. During the encoding task, 60 personality trait words, half of which are unambiguously positive, will be presented each for 500 ms and participants will be requested to indicate whether the word is likeable or dislikeable in the context of this word being used to describe them. Each word will be preceded by a black fixation cross centrally presented against a solid gray background for 500 ms. Words will be followed by a jittered ITI following a Poisson distribution ranging from 1 to 6 seconds (mean of 3 seconds) where a centrally-presented black fixation cross will appear against a solid gray background. For the memory retrieval task, 120 personality trait words will be presented, of which half will be repeated from the categorisation task and half will be new words (distractors) comprised of an equal number of unambiguously positive and unambiguously negative distractor words. Participants will be asked to indicate whether a presented word is new (not seen before) or old (seen before).

**fMRI data acquisition–encoding and retrieval task** The scan length is approximately 13 minutes, during which 384 volumes of fMRI data will be collected.

**Resting state** Participants will be asked to lie at rest with their eyes closed and try not to fall asleep during continuous fMRI data acquisition.

**fMRI data acquisition–resting state** The scan length is approximately 9 minutes, during which 265 volumes of fMRI data will be collected. To measure the magnetic field prior to and following resting state data acquisition field maps with two echo times (TE1 = 5.19 ms, TE2 = 7.65 ms) will also be acquired with the positioning of the participant kept consistent across the three scan sequences. Magnitude images for both echoes and estimated field maps will be obtained.

### Follow-up assessment

Patients who were randomized to the CBT arm of the IMPACT clinical trial who have completed a minimum of 6 out of a maximum of 20 offered CBT therapy sessions will be invited back for a second MRI visit approximately 24 weeks following their first therapy appointment. All healthy comparison participants will be invited back for a second MRI visit at least 24 weeks from their first scan date to ensure a comparable length of time has passed between scans across groups. Due to financial constraints, no sMRI and DTI sequences will be performed at the second MRI visit. All participants will complete the same fMRI tasks collected during the first MRI visit (e.g., implicit facial expression recognition, emotional go/no go, encoding and retrieval, and resting state tasks). In line with procedures undertaken at the first scan, STAI measures will be obtained prior to scanning whereas SMFQ and Bond VAS measures will be obtained after the scan.

### Sample size and power calculations

To effectively test the presence of a relationship between pre-treatment imaging markers and post-treatment symptomatic response the study sample size will need to be large enough to overcome the neuroimaging problem of multiple comparisons across the ~300,000 intra-cerebral image voxels and also account for participant attrition. The attrition rate observed during a similar trial (i.e., Adolescent Depression Antidepressants and Psychotherapy Trial [ADAPT]) with a 28-week follow-up period was just 2% [43]. Allowing for an even greater attrition rate of 20% on a pre-treatment sample size of 40 patients per treatment arm, it follows that 32 patients will be available for post-treatment scans. Assuming an effect size of the same size as that observed in a previous study of adults with MDD (R ≥ 0.76 for grey matter volume [GMV] and symptom response in ACC) [8] and a conservative two-tailed probability of p <0.001 to control for multiple comparisons in a whole brain analysis of imaging data, and at least 32 patients completing post-treatment scans, the power to detect a true effect will be at least 95%. Thus with pre-treatment imaging data from 40 patients per treatment arm, the study is strongly powered to assess imaging predictors of recovery following treatment for MDD.

To detect CBT-related changes in brain function, a previous fMRI study of adult MDD [9] and a pilot study examining components of variance in a facial emotion perception task [44] are used to inform calculations of statistical power. Activation of the left amygdala by facial emotion processing showed an effect of antidepressant treatment of d = 1.02 [9]. A similar effect size of CBT will be assumed for the current study, with empirical estimates of between-subject and between-session variance in amygdala response [44] suggesting that a minimum power of 80% will be needed for detection of a true treatment effect. Considered together with a false positive rate of 5%, a minimum of 30 participants will be needed for the detection of CBT-related changes in brain function. The effects obtained in 40 patients in the CBT arm, compared to 40 healthy adolescents, should therefore be sufficiently powerful in size to detect therapeutic changes in amygdala activation equivalent to that observed from antidepressant medication treatment studies of adult patients with MDD.

#### *sMRI data analysis plan*

Whole brain, multi-channel segmentation [45] and voxel-based morphometry [46] will be used to generate GMV maps from each baseline scan for co-registration into Montreal Neurological Institute (MNI) standard space [47]. Whole brain voxel-based morphometry will first be used to assess the presence of pre-treatment anatomical differences between patients and controls and to explore regions where variation in grey or white matter significantly predict treatment-specific or treatment-general responses [8]. The multivariate technique of partial least squares [48] will also be used to identify patterns of grey matter variation that optimally correlate with treatment response. In an effort to test our hypotheses comprehensively, we will also use the MNI template [49] to anatomically parcellate a priori regions of interest including, but not limited to, subgenual, pregenual and dorsal ACC, amygdala, hippocampus, and prefrontal cortex, where the presence of both group differences in GMV and associations with treatment-specific symptom reductions will be assessed. Non-parametric statistical methods with correction for multiple comparisons [50-52] will be used.

With regard to the DTI dataset, fractional anisotropy and mean diffusivity maps will be created using the Functional Magnetic Resonance Imaging of the Brain (FMRIB) diffusion toolbox in FMRIB Software Library (FSL) [53] by estimating diffusion tensors at each intracerebral voxel prior to using a b = 0 image for mapping into MNI standard space. As with the sMRI data, between-group differences and predictors of treatment response in fractional anisotropy and diffusivity measures will be identified across the whole brain and in a priori regions of interest. For further exploration of group differences and associations with treatment outcome, anatomical connections between frontal and limbic cortex will be measured using probabilistic tractography [54-56].

#### *fMRI data analysis plan*

Standard preprocessing methods for all datasets will include correction for head movement [52] and appropriate control for potential confounds to BOLD signal dynamics such as fluctuation in cardiorespiratory rhythms [57]. A general linear model will be used to convolve the hemodynamic response function with fMRI-related contrasts of interest to provide estimates of BOLD signal on a voxel-by-voxel basis in MNI standard space. Hypotheses will be tested using the same approach as that undertaken in the sMRI data analyses, where between-group differences and predictors of treatment response in pretreatment BOLD signal will be identified across the whole brain and in a priori regions of interest. CBT-related changes in functional activation will also be examined. Univariate analyses will be followed-up by the multivariate approach of partial least squares to identify task-related patterns of functional activation relating to therapeutic outcome.

Resting state fMRI data will be analysed in a similar fashion to the functional task data. The Hurst exponent will first be estimated at each voxel in the resting state data. The Hurst exponent measures the randomness of a data set and as such, has been shown to be sensitive to the effects of disease and drug treatment [58,59]. Between-group differences and predictors of treatment response in pretreatment BOLD signal will first be identified across the whole brain and then in a priori regions of interest. CBT-related changes in the Hurst exponent will also be examined.

We will also measure functional connectivity between regions within the fronto-limbic system and construct graphical models that summarise the topological properties of fronto-limbic and whole brain networks [60,61] in the resting state data. Similar network analysis methods may then be applied to the DTI data set, thereby permitting an integrated functional and structural analysis of network abnormalities in depression. Nonparametric statistical methods of inference will again be applied to statistically evaluate fractal and network parameters [50-52,62].

#### *Longitudinal data analysis plan*

For the fMRI datasets collected at baseline and 24 weeks, we will fit a balanced, mixed effects 2 × 2 (group × time) analysis of variance (ANOVA) model to the brain activation data acquired on patients treated with CBT and healthy adolescents. A significant group × time interaction will identify the effect of CBT, for which appropriate post-hoc tests will be performed. Nonparametric statistical methods of inference correcting for multiple comparisons will be applied to statistically evaluate the main effects of group and time point, and any group × time interaction [50-52].

## Discussion

An important challenge to adolescent mental health services is the provision of appropriate and effective treatment to adolescents in need at the earliest possible point in time. To overcome this challenge, accuracy in both diagnostic assessment and prognostic evaluation of patients are critical, but as yet clinicians possess no objective tools for either. Diagnostic assessments rely on subjective retrospective information transmission from patient and/or parent to clinician whereas potential prognostic indicators of treatment outcome, such as depressive symptom severity or suicidal tendencies, are poor [43]. Therefore the overall aim of MR-IMPACT is to identify and evaluate objective neurobiological markers of depressive illness in adolescence and prognostic indicators of recovery following psychological treatment for depression.

Neural markers of depression in adolescence will be characterized by comparing structural and functional differences in patients with MDD prior to treatment against a group of healthy adolescent participants matched for demographic characteristics such as age, sex and IQ. Treatment-related changes will be identified by measuring changes in brain functional activation and connectivity prior to treatment and following at least six attended sessions of CBT. A placebo-controlled trial would have been an optimal method for assessing treatment-related changes, but impossible due to ethical constraints. Therefore, changes in brain function in healthy adolescents will also be measured at a second time point matched in length to that of the depressed group to ensure that functional changes indicative of adolescent development are not misidentified as changes related to CBT treatment.

Short, medium, and long-term predictors of response to treatment by CBT, STPP, and SCC will be identified by relating the extent and rate of improvement in depressive symptoms with pre-treatment measures of brain structure, functional activity and connectivity. To comprehensively test the relationship between structural and functional brain measures and the clinical phenotypes of the depressions we will also perform multivariate analyses focusing on neural systems implicated in adult MDD and on the fronto-limbic system in particular. The longer-term aim of MR-IMPACT, IMPACT, and other adjunct IMPACT studies includes whether relapse and recurrence of depression can be successfully predicted using neurobiological, behavioural, environmental, and genetic indices.

Despite the aim of differentiating state from trait markers of depressive illnesses in adolescence and predictors treatment response, our study is not without limitations. One limitation, common to all longitudinal treatment studies, includes an inability to disaggregate neural markers of illness and neural indicators of treatment-general non-response (i.e., refractory depression). While the simple comparison of treatment responders with treatment non-responders would indicate regions involved in treatment-specific response, the possibility remains that patients would have responded to alternative treatment strategies had they been provided. One way we could have examined neural markers of illness would have been to include within the longitudinal design a group of currently healthy adolescents with a predisposition for developing MDD. However, in order to achieve sufficient statistical power to ensure reliable and reproducible effects, this would have necessitated the initial recruitment of a much larger sample of adolescents than was realistically feasible and so we opted for the current design. Bearing this limitation in mind, we hope the study results will assist in the provision of more targeted treatment strategies to adolescent patients with MDD.

## Abbreviations

3D-MPRAGE: 3-dimensional magnetically prepared rapid acquisition gradient echo; ACC: Anterior cingulate cortex; ADAPT: Adolescent depression antidepressants and psychotherapy trial; ANOVA: Analysis of variance; BOLD: Blood oxygen level-dependent; CAMHS: Child and Adolescent Mental Health Services; CBT: Cognitive behavioural therapy; CDI: Children’s depression inventory; CSPTC: Cortico-striato-pallidal-thalamo-cortical; dlPFC: Dorsolateral prefrontal cortex; DISC: Diagnostic interview schedule for children; DSM-IV: Diagnostic and statistical manual for mental disorders, fourth edition; DTI: Diffusion tensor imaging; EPI: Echo planar imaging; FEEST: Facial expressions of emotion stimuli and tests; fMRI: Functional magnetic resonance imaging; FMRIB: Functional magnetic resonance imaging of the brain; FSL: FMRIB software imaging laboratory; GMV: Grey matter volume; HPA: Hypothalamic-pituitary-adrenal; IMPACT: Improving mood with psychoanalytic and cognitive therapies; IQ: Intelligence quotient; ITI: Inter-trial-interval; KSADS-PL: Kiddie schedule of affective disorders and schizophrenia- present and lifetime version; MDD: Major depressive disorders; MFQ: Mood and feelings questionnaire; MNI: Montreal Neurological Institute; MRI: Magnetic resonance imaging; MR-IMPACT: Magnetic resonance: improving mood with psychoanalytic and cognitive therapies; NHS: National Health Service; NICE: National Institute for Health and Care Excellence; OFC: Orbitofrontal cortex; PD-T2: Proton density T2-weighted; pgACC: Pregenual anterior cingulate cortex; ROI: Region of interest; SCC: Specialist clinical care; SMFQ: Moods and feelings questionnaire–short version; sMRI: Structural magnetic resonance imaging; SSRI: Selective serotonin reuptake inhibitor; STAI-S: Spielberger trait anxiety inventory-state; STAI-T: Spielberger trait anxiety inventory-trait; STPP: Short-term psychoanalytic psychotherapy; sgACC: Subgenual anterior cingulate cortex; TE: Echo time; TR: Repetition time; UK: United Kingdom; VAS: Visual analogue scales.

## Competing interests

Professor Bullmore is a part-time employee of GlaxoSmith Kline. Professor Sahakian consults for Cambridge Cognition, Lundbeck and Servier. Professor Sahakian also holds a grant from Janssen/J&J and owns share options in Cambridge Cognition, LLP. Professors Fonagy, Goodyer, Reynolds, Target and Suckling, and Drs. Hagan, Holt, Kelvin, Lennox, Ooi and Wilkinson report no competing interests. Ms. Graham and van Nieuwenhuizen and Mr. Widmer similarly report no competing interests.

## Authors’ contributions

CH wrote the manuscript and led the manuscript development. All authors edited the manuscript and approved the final version. CH is the Project Coordinator for the study. JS conceived the project, led the study development process and is Principal Investigator of the study. IG contributed to study development and is Chief Investigator of the IMPACT study.

## Pre-publication history

The pre-publication history for this paper can be accessed here:

http://www.biomedcentral.com/1471-244X/13/247/prepub
